# Infolding of Self-Expandable Transcatheter Heart Valve: Case Report and Review of Literature

**DOI:** 10.7759/cureus.10093

**Published:** 2020-08-28

**Authors:** Wassef Karrowni, Saleh Fakih, Pierre Nassar

**Affiliations:** 1 Cardiovascular Disease, UnityPoint Health, Cedar Rapids, USA; 2 Cardiovascular Disease, Lebanese University, Beirut, LBN; 3 Cardiovascular Disease, Beirut Cardiac Institute, Beirut, LBN

**Keywords:** transcatheter aortic valve replacement, infolding

## Abstract

Transcutaneous aortic valve replacement (TAVR) has become a widely accepted minimally invasive approach for treatment of severe aortic stenosis. Self-expandable prostheses are commonly the device of choice, with excellent procedural success and durability. However, there have been several recent case reports of infolding of the self-expandable prosthesis during development with subsequent malfunction and need for further intervention. We present a case of self-expandable valve prosthesis infolding managed by balloon postdilation, and summarize the cases reported in the literature to date in an attempt to increase awareness of this serious technical problem and the factors associated with it.

## Introduction

Transcatheter aortic valve replacement (TAVR) is now a well-established therapy for patients with symptomatic severe aortic stenosis. It has been proven in randomized controlled studies to be non-inferior compared with surgical aortic valve replacement (SAVR) among patients with low, intermediate, and high surgical risk [1-4]. The most commonly used TAVR prostheses are either balloon-expandable or self-expandable in design. Both designs can be used interchangeably in most clinical situations, however, each one has its own advantages and disadvantages. TAVR using self-expandable prosthesis has demonstrated excellent procedural success and durability, but one of its pitfalls is the rare occurrence of infolding of the frame during deployment with secondary distortion and malfunction of the prosthesis. Infolding of self-expandable valve prosthesis during TAVR is an under-reported and under-recognized phenomenon. Herein, we report a case of severe infolding, review the literature of reported cases, and discuss the identified risk factors and approaches for management.

## Case presentation

**Figure 1:**
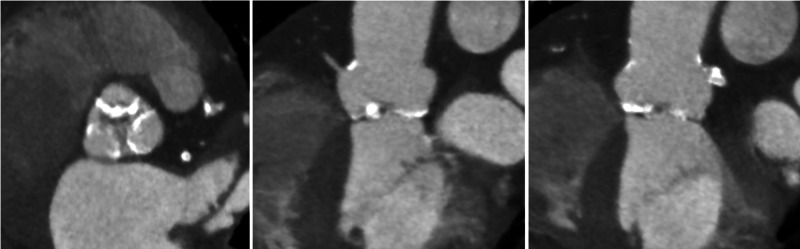
Multislice computed tomographic scan showing heavily calcified trileaflet aortic valve.

An 85-year-old gentleman with symptomatic severe aortic stenosis was referred for TAVR. He had history of prior coronary artery bypass grafting (CABG). Transthoracic echocardiogram revealed an ejection fraction of 35%, aortic valve area estimated at 0.5 cm^2^, and a mean gradient of 52 mmhg; there was also moderate aortic insufficiency. Cardiac computed tomography demonstrated heavily calcified valve with estimated annulus perimeter of 86 mm consistent with a 34-mm Evolut R (Medtronic Inc., Minneapolis, MN) valve (Figure 1).

**Figure 2:**
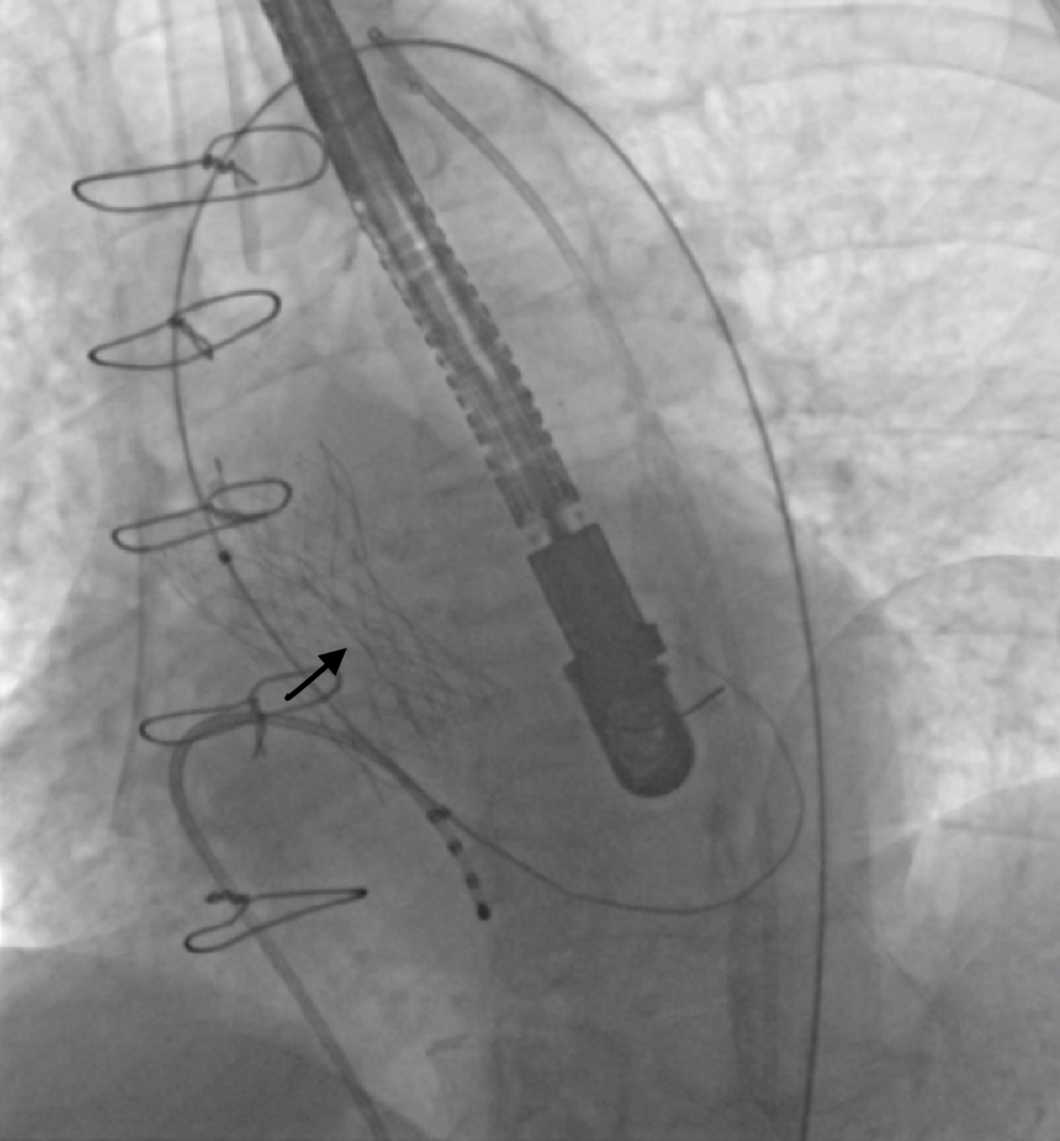
Straight-line distortion of the valve cells. The vertical or straight-line distortion (arrow) of the valve cells indicating infolding of a 34-mm Evolut R valve (Medtronic Inc., Minneapolis, MN).

The Evolut R was advanced through in-line sheath in the right transfemoral access. First deployment at the two-thirds position demonstrated a high implant. The valve was recaptured and redeployed to optimal depth but the inflow part of the valve appeared to be under-expanded and with the appearance of a vertical line in the axis of the valve; this was persistent after the valve was fully released (Figure 2). Transesophageal echo (TEE) revealed severe aortic insufficiency.

Post-dilation was performed initially with 27-mm balloon with disappearance of the distortion (Video 1).

**Video 1:** Post-dilation of Evolut R valve with significant improvement in valve expansion.

Repeat TEE images revealed only trace paravalvular leak.

## Discussion

Several cases of infolding of self-expandable valves have been reported in the literature [5-11]. The true incidence is not clear but it is believed to be of rare occurrence with an estimate of 3.15% based on a recent report [12]. Its occurrence is related to the low radial force of the nitinol frame of the self-expandable valve. Several risk factors for this phenomenon have been identified, some of which are related to the anatomy of the patient and some are technique and others are device related (Table 1) [5-11].

**Table 1:** Reported risk factors for self-expandable valve infolding.

Anatomical Factors	Device-Related Factors
Eccentric and/or heavy calcifications	Improper loading
High ellipticity of annulus	Larger prosthesis sizes (29 mm or larger)
Bicuspid native valve	Re-sheathing

The most common clinical scenario for occurrence of infolding is in the patients who require large valve sizes (29 mm or larger) and in combination with significant valve calcifications. Careful fluoroscopic inspection, sometimes rotational, of the valve could help detect this serious complication [9]. The deformation is usually associated with significant aortic insufficiency and/or stenosis from the malfunction of one of the leaflets. The management is either post-dilation of appropriately sized balloon, or retrieval and deployment of a new valve. In our case, after the first post-dilation there was only partial correction of the infolding with change in the fluoroscopic appearance from a vertical line to distortion limited to the level of the annulus. This required another post-dilation with a larger and more appropriately sized balloon with full correction of the problem.

In Table 2, we summarize the relevant cases published in the literature to date. All the reported cases were with the different versions of the Medtronic self-expandable valve (CoreValve, Evolut R, and Evolut Pro). The prosthesis’ sizes in the incident cases were 29 mm or larger. Most of the patients did have balloon pre-dilation done which argues against pre-dilation as preventive strategy for prosthesis infolding. Resheathing of the prosthesis for improper depth of first implant was of frequent occurrence (half of the cases). Six of eight cases were managed successfully with balloon post dilation and the remaining two with retrieval and deployment of a new device (one with Edwards Sapien 3 and one with same original device of Evolut Pro). No adverse in-hospital clinical events were reported in this series of patients. However, a recent study reported an alarming number of non-disabling stroke occurring in association with infolding of self-expandable valves [12].

**Table 2:** Summary of cases of infolding of self-expandable valves published in literature.

Case Report	Type	Size	Pre-dilation	Resheathing	Management	Paravalvular Leak
Sinning et al. [5]	CoreValve	29-mm	Yes	No	Post-dilation 28-mm Balloon	Mild
Kaple et al. [6]	CoreValve	31-mm	Yes	No	Post-dilation 25-mm Balloon	None
Ben-Dor et al. [7]	Evolut Pro	29-mm	Yes	Yes	Post-dilation 24-mm Balloon	None
Abdelghani et al. [8]	Evolut R	34-mm	Yes	Yes	Device removed and Edwards Sapien 3 implanted	None
Kagase et al. [9]	Evolut Pro	29-mm	Yes	Yes	Device retrieved and another Evolut Pro 29-mm implanted	Trace
Wiper et al. [10]	CoreValve	29-mm	Yes	No	Post-dilation 25-mm Balloon	Moderate
Kataoka et al. [11]	Evolut Pro	29-mm	No	No	Post-dilation 20-mm Balloon	None
Our Case	Evolut R	34-mm	No	Yes	Post-dilation 27-mm Balloon	Trace

## Conclusions

Infolding of self-expandable valves is an underrecognized complication of TAVR by the structural interventionalists that is associated with several anatomical and device-related factors. There is need for more research and data collection to further evaluate the best approach to manage it and whether it is associated with any adverse clinical events.

## References

[1] Adams DH, Popma JJ, Reardon MJ (2014). Transcatheter aortic-valve replacement with a self-expanding prosthesis. N Engl J Med.

[2] Leon MB, Smith CR, Mack MJ (2016). Transcatheter or surgical aortic-valve replacement in intermediate-risk patients. N Engl J Med.

[3] Reardon MJ, Van Mieghem NM, Popma JJ (2017). Surgical or transcatheter aortic-valve replacement in intermediate-risk patients. N Engl J Med.

[4] Popma JJ, Deeb GM, Yakubov SJ (2019). Transcatheter aortic-valve replacement with a self-expanding valve in low-risk patients. N Engl J Med.

[5] Sinning JM, Vasa-Nicotera M, Ghanem A, Grube E, Nickenig G, Werner N (2012). An exceptional case of frame underexpansion with a self-expandable transcatheter heart valve despite predilation. JACC Cardiovasc Interv.

[6] Kaple RK, Salemi A, Wong SC (2017). Balloon valvuloplasty treatment of an infolded CoreValve. Catheter Cardiovasc Interv.

[7] Ben-Dor I, Rogers T, Satler LF, Waksman R (2019). A word of caution using self-expanding transcatheter aortic valve-frame infolding. Catheter Cardiovasc Interv.

[8] Abdelghani M, El Ghalban A, Landt M, Richardt G, Abdel-Wahab M (2018). In vivo stent frame infolding of a self-expanding transcatheter aortic valve after resheathing. JACC Cardiovasc Interv.

[9] Kagase A, Yamamoto M, Nishio H, Tsujimoto S (2019). Importance of rotational angiography before complete release of self-expandable transcatheter bioprosthesis for detecting valve infolding phenomenon. JACC Cardiovasc Interv.

[10] Wiper A, Chauhan A, More R, Roberts D (2014). CoreValve frame distortion: the importance of meticulous valve loading. JACC Cardiovasc Interv.

[11] Kataoka A, Watanabe Y, Nagura F (2018). Balloon valvuloplasty for Evolut R infolding: useful transesophageal echocardiographic monitoring for diagnosis and efficacy. JACC Cardiovasc Interv.

[12] Musallam A, Rogers T, Ben-Dor I, Torguson R, Khan JM, Satler LF, Waksman R (2020). Self-expanding transcatheter aortic valve-frame infolding: a case series with a warning message. JACC Cardiovasc Interv.

